# Data on *in vivo* selection of SK-OV-3 Luc ovarian cancer cells and intraperitoneal tumor formation with low inoculation numbers

**DOI:** 10.1016/j.dib.2015.12.037

**Published:** 2016-01-06

**Authors:** Elly De Vlieghere, Charlotte Carlier, Wim Ceelen, Marc Bracke, Olivier De Wever

**Affiliations:** a Laboratory of Experimental Cancer Research, Ghent University, Belgium; b Laboratory of Experimental Surgery, Ghent University hospital, Belgium

**Keywords:** *in vivo* selection, Ovarian cancer, Mouse model, Growth curves, Survival curves

## Abstract

This data paper contains information about the *in vivo* model for peritoneal implants used in the paper “Tumor-environment biomimetics delay peritoneal metastasis formation by deceiving and redirecting disseminated cancer cells” (De Vlieghere et al., 2015) [1]. A double *in vivo* selection of SK-OV-3 Luc human ovarian cancer cell line was used to create SK-OV-3 Luc IP1 and SK-OV-3 Luc IP2 cell lines. This data paper shows functional activities of the three cell lines *in vitro* and *in vivo.* Phase-contrast images show the morphology of these cells, metabolic and luciferase activity has been determined. Survival data of mice peritoneally injected with SK-OV-3 Luc or SK-OV-3 Luc IP2 is available with H&E histology of the peritoneal implants. Tumor growth curves and bioluminescent images of mice inoculated with a different number of SK-OV-3 Luc IP2 cells are also included.

**Specifications table**

**Table:** 

Subject area	*Biology*
More specific subject area	*Cancer biology*
Type of data	*Phase contrast images, graphs, histology, bioluminescence images*
How data was acquired	• *Phase contrast microscope ( DMI 3000B,Leica, Wetzlar, Germany)* • *Plate reader (Paradigm, Molecular Devices, Sunnyvale, CA, USA)* • *Light microscope (DM750,Leica, Wetzlar, Germany)* • *Bioluminescent imager (IVIS, PerklinElmer, Waltham, MA, USA)*
Data format	*Analyzed*
Experimental factors	SK-OV-3-Luc cells were inoculated intraperitoneally in immune deficient female mice Swiss/nu to select for a population that more efficiently forms peritoneal implants.
Experimental features	The created cell line SK-OV-3 Luc IP1 and SK-OV-3 Luc IP2 are compared with the parental cell line SK-OV-3 Luc
Data source location	*Ghent, Belgium*
Data accessibility	*The data is available with this article*

**Value of the data**

• This data shows intraperitoneal tumor formation with low cell inoculation after *in vivo* selection of SK-OV-3.
• This method can be applied to other cancer cell lines to increase metastasis take rate even with lower inoculation numbers.
• These data provides growth curves, survival data and histology about the SK-OV-3 Luc (IP2) *in vivo* model for peritoneal implants, providing researchers with a references for their *in vivo* studies.

## 1 Data

Intraperitoneal injection (IP) of SK-OV-3 (Luc) cells is an established *in vivo* model for the development of peritoneal ovarian tumor implants. Usually inoculation numbers of 1–2×10^6^ SK-OV-3 (Luc) are used 2, 3, 4. The IP injection of 1×10^6^ SK-OV-3 Luc cells gives a 100% tumor take with an average survival time of over 2 months (Fig. 3B) [3]. To better mimic the patient situation where an initial low number of aggressive disseminated cells can form extensive peritoneal metastasis a xenograft mouse model with inoculation of low numbers of cancer cells is needed. *in vivo* selection has been successfully used to increase the metastatic potential of MDA-MB-231 [5]. These data describe the successive *in vivo* selection of the SK-OV-3 Luc cell line. Tumor implants of SK-OV-3 Luc bearing mice are re-cultured *in vitro* to increase *in vivo* peritoneal implant formation. After successive *in vivo* selection, IP inoculation of 10-to-20X lower number (1×10^5^) of SK-OV-3 Luc IP2 is sufficient to form peritoneal implants this model has been applied successfully [1].

## 2 Experimental design, materials and methods

### 2.1 Cell culture conditions

SK-OV-3 is a human ovarian cancer cell line (ATCC number: HTB-77). SK-OV-3 Luc (Luciferase positive SK-OV-3 cells) were prepared by pFL4.76 plasmid transfection and selection (Promega, Leiden, The Nederlands). SK-OV-3 Luc (IP1/2) were maintained in Dulbecco’s modified Eagle’s medium (DMEM) supplemented with 10% fetal bovine serum (FBS) and antibiotics (penicillin/streptomycin), and incubated at 37 °C with 10% CO_2_ in air.

### 2.2 *in vivo* selection

**Fig. 1:**
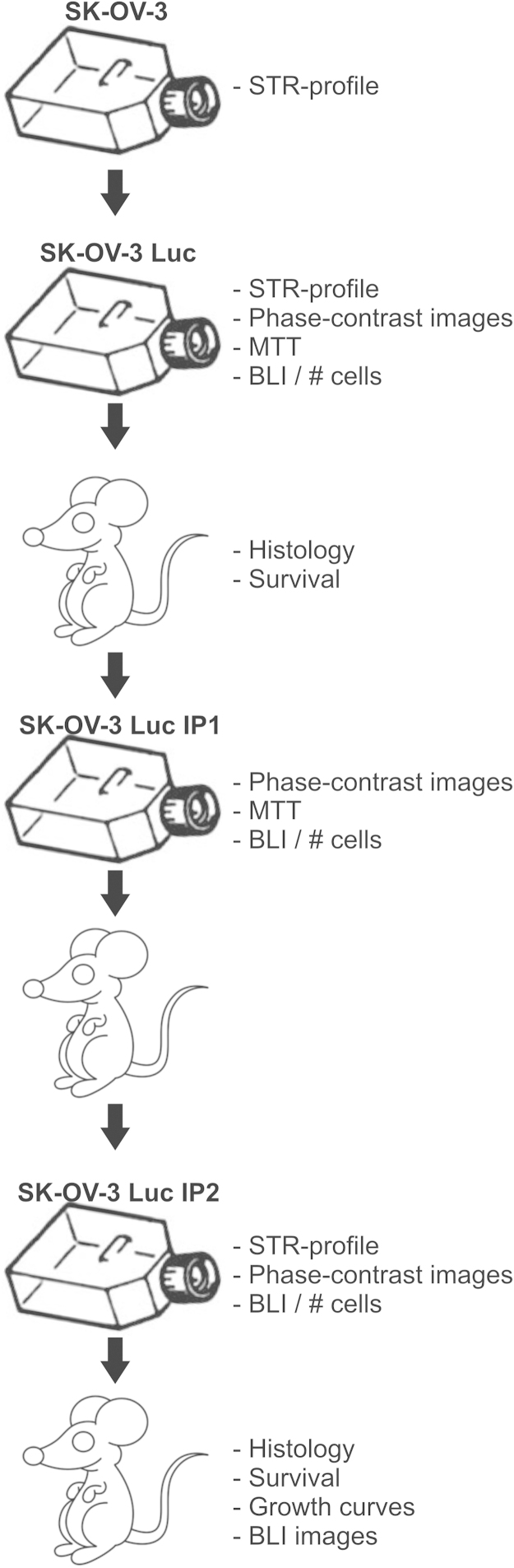
Schematic showing the successive stages of the *in vivo* selection with indication of the different *in vitro* and *in vivo* assays conducted.

**Fig. 2:**
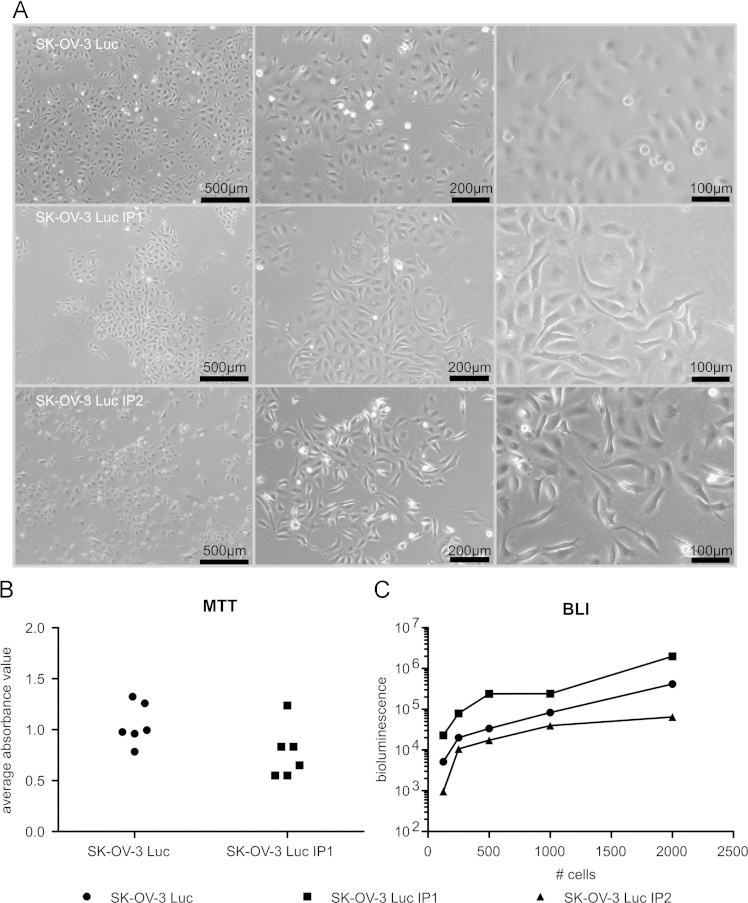
(A) Phase-contrast images of SK-OV-3 Luc, SK-OV-3 Luc IP1 and SK-OV-3-Luc-IP2 cultured cell cultured treated culture flasks. (B) Metabolic activity analysis (MTT): the average absorbance value (*n*=3) of 6 independent metabolic activity analysis 96 h after seeding 4×10^4^ SK-OV-3 Luc or SK-OV-3 Luc IP1. (C) *in vitro* average bioluminescence (*n*=3) of SK-OV-3 Luc, SK-OV-3 Luc IP1 and SK-OV-3-Luc-IP2 for 125, 250, 500, 1000 and 2000 cells, 2 h after seeding.

Animal experiments were conducted accordance with the local ethics committee (Ghent University Hospital). 1×10^6^ SK-OV-3-Luc cells were inoculated intraperitoneally in immunodeficient female Swiss/nu mice (Charles River, Chatillon-sur-Chalaronne, France) to isolate populations that form peritoneal implants. Tumor implants were collected in saline. The implants were cut into small pieces (1 mm) and dissociated by de gentleMACS Dissociator (Miltenyi Biotec, Teterow, Germany) in the presences of 1 mg/ml collagenase from Clostridium histolyticum (Sigma-Aldrich) in PBSD^+^. The mixture was cleared through a cell strainer (70 µm) and the homogenized single cell suspension was expanded in culture by seeding in DMEM 10% FBS. After 24 h, non-adhered cells were removed and the medium was refreshed; the resulting culture was maintained as the parental cell line. Each subsequent intraperitoneal metastatic generation is designated IP1, IP2. Fig. 1 shows a schematic with the different stages of the *in vivo* selection. Fig. 2A shows phase-contrast images of the three cell lines cultured on plastic

### 2.3 Sort tandem repeat (SRT) profiling

SRT-profiling was conducted on the SK-OV-3, SK-OV-3 Luc and SK-OV-3 Luc IP2 cell lines to confirm the identity of the cell lines. 9 DNA sites were profiled and the alleles were identical between the three cell lines and to the profile published by ATCC (Amelogenin: X; CSF1PO: 11; D13S317: 8,11; D16S539: 12; D5S818: 11; D7S820: 13,14; THO1: 9,9.3; TPOX: 8,11; vWA: 17,18) [6].

### 2.4 Metabolic activity analysis: 3-4,5-Dimethylthiazolyl-2-2,5-Diphenyltetrazolium Bromide (MTT)

A single cell suspension of 4×10^4^ SK-OV-3 Luc or SK-OV-3 Luc IP1 cells per well, were seeded in a 96-well plate. After 96 h, MTT analysis was performed. Briefly, the culture medium was replaced by 100 μl culture medium containing 1 mg/ml MTT. Following 2 h incubation at 37 °C, MTT-containing medium was removed and 150 μl of dimethylsulfoxide (DMSO) was added to dissolve formazan crystals. Absorbance was measured at 570 nm and background measured at 650 nm, with a plate reader (Paradigm, Molecular Devices). Fig. 2B shows the average absorbance value (*n*=3) of 6 independent metabolic activity analysis 96 h after seeding 4×10^4^ SK-OV-3 Luc or SK-OV-3 Luc IP1.

### 2.5 *in vitro* bioluminescence

A single cell suspension of SK-OV-3 Luc, SK-OV-3 Luc IP1 and SK-OV-3-Luc-IP2 cells were seeded in a 96-well plate in different cell numbers (125, 250, 500, 1000 and 2000 cells per well). Cells were allowed to adhere for 2 h, just before luciferase activity was measured with IVIS (PerkinElmer), 150 µg/ml D-Luciferin, firefly (Perkin-Elmer) was added. Fig. 2C shows the average bioluminescence value of three replicates.

### 2.6 *in vivo* survival analysis

**Fig. 3:**
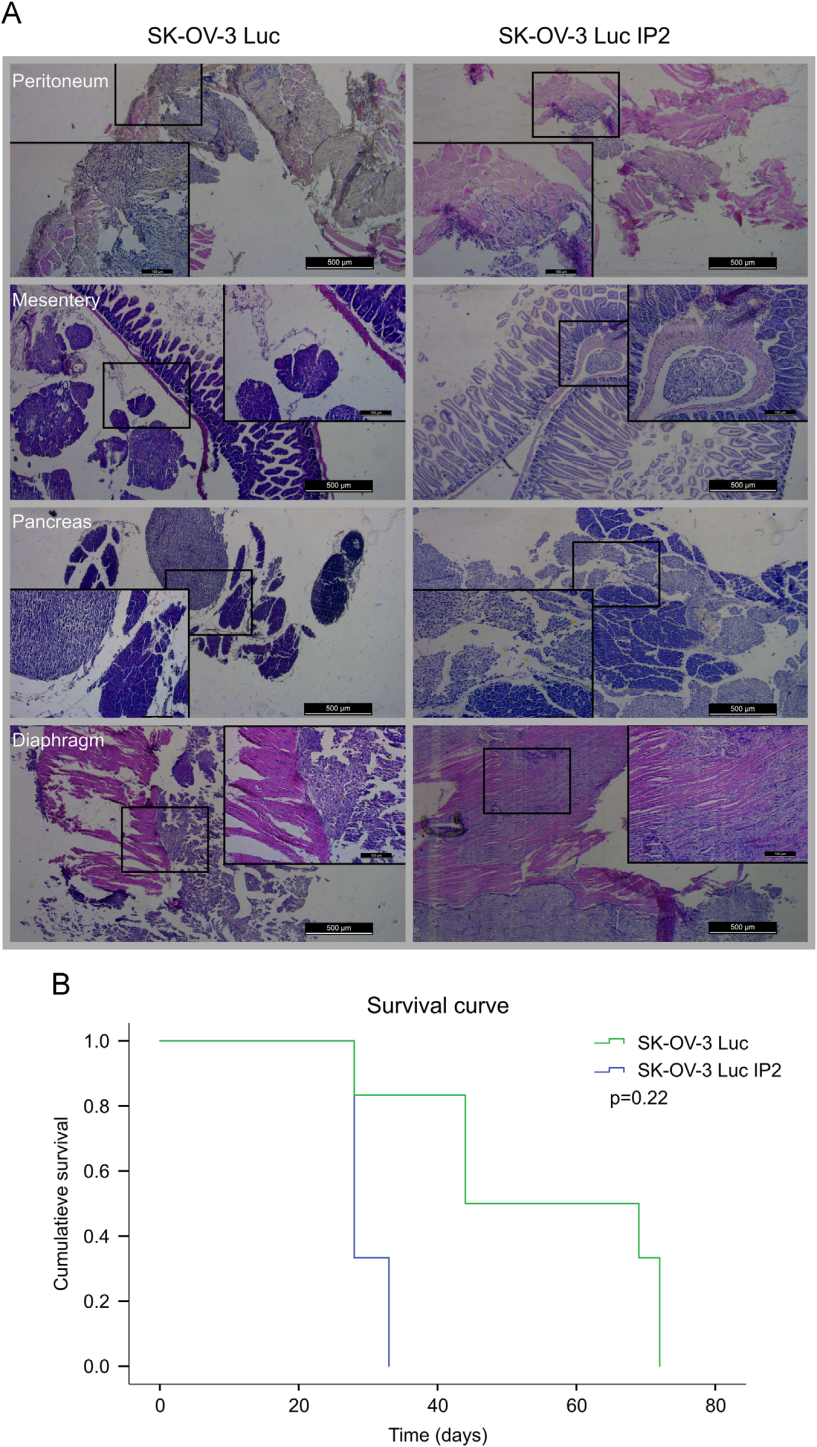
(A) H&E staining׳s of peritoneal implants at the site of peritoneum, mesentery, pancreas and diaphragm of SK-OV-3 Luc or SK-OV-3 Luc IP2 tumors. (B) Comparative survival curves of mice IP injected with 1×10^6^ SK-OV-3 Luc and SK-OV-3 Luc IP2 cells.

Animal experiments were conducted in accordance with the local ethics committee (Ghent University Hospital). Immune deficient female Swiss/nu mice were inoculated intraperitoneally with 1×10^6^ SK-OV-3-Luc or SK-OV-3-Luc-IP2 cells. Mice were monitored and from the first visual signs of advanced carcinomatosis (decrease in weight or increase in abdominal circumference) the mice had reached their end-point and were sacrificed. Peritoneal organs were embedded in paraffin before standard hematoxylin and eosin (H&E) staining was conducted. Fig. 3A shows H&E staining of the peritoneal tumor implants, B shows comparative survival curves.

### 2.7 *in vivo* growth curves

**Fig. 4:**
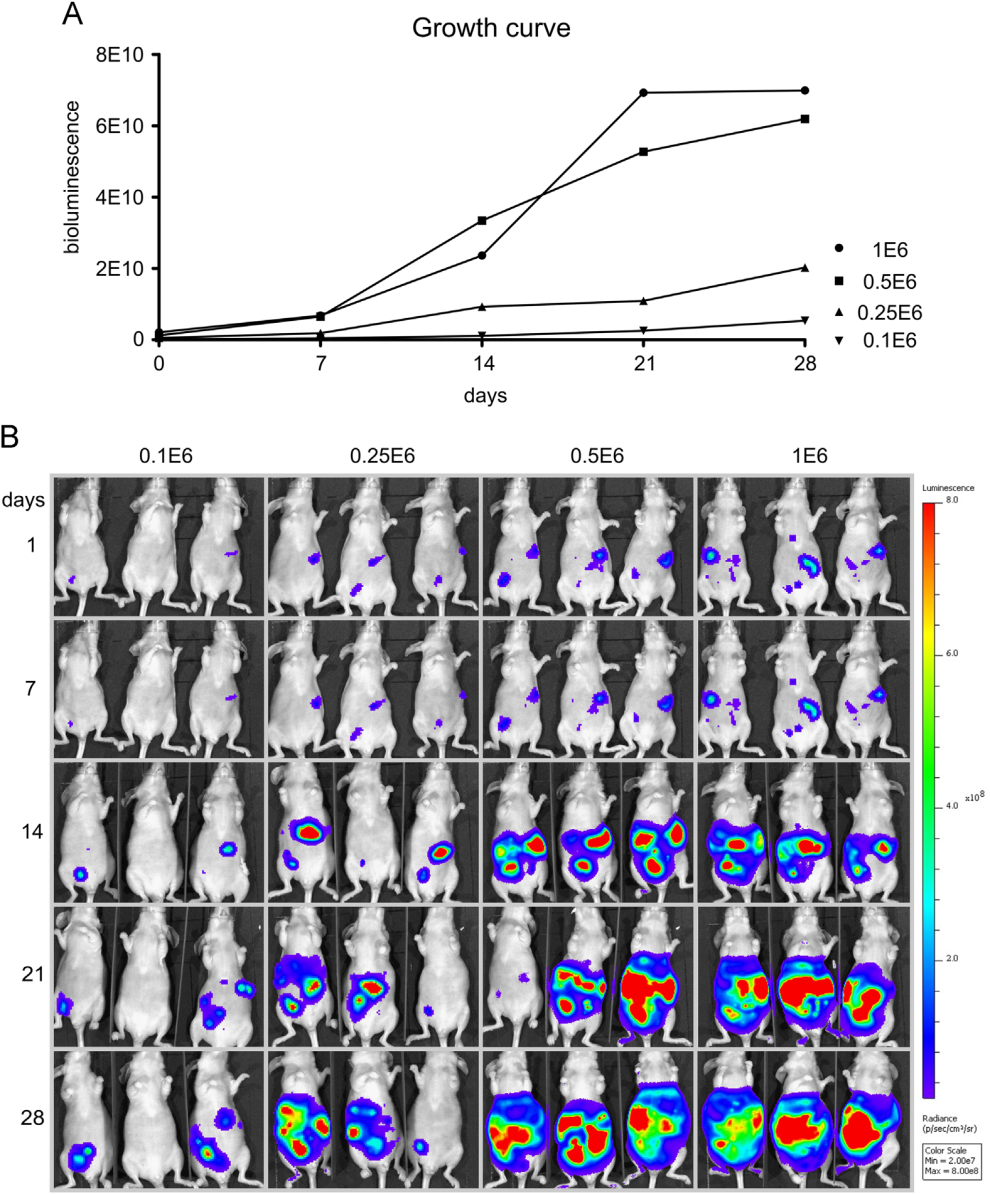
Growth curves (A) and BLI (B) of mice inoculated with 1×10^6^, 0.5×10^6^, 0.25×10^6^ or 0.1×10^6^ SK-OV-3 Luc IP2 cells (*n*=3).

Immune deficient female Swiss/nu mice were inoculated intraperitoneally with 1×10^6^, 0.5×10^6^, 0.25×10^6^ and 0.1×10^6^ SK-OV-3 Luc IP2 cancer cells. Tumor growth was monitored by weekly bioluminescence imaging (BLI). Fig. 4 shows the different growth curves (A) and the bioluminescence images (B).
